# Supplementary material for: 3D printing of self-healing personalized liver models for surgical training and preoperative planning
Source: Nat Commun. 2023 Dec 19;14:8447. doi: 10.1038/s41467-023-44324-6 (PMC10730511; doi:10.1038/s41467-023-44324-6)
Supplement: Supplementary file 1 — Supplementary Information [file 41467_2023_44324_MOESM1_ESM.pdf]

Supplementary Information

3D printing of self-healing personalized liver models for surgical training and preoperative planning

Yahui Lu[1†], Xing Chen[2,3†], Fang Han[2,3], Qian Zhao[1], Tao Xie[1], Jingjun Wu[1,4,*], Yuhua Zhang[2,3*]

[1]State Key Laboratory of Chemical Engineering, College of Chemical and Biological Engineering, Zhejiang University, Hangzhou 310027, China

[2]Zhejiang Cancer Hospital, Hangzhou, Zhejiang 310022, China

[3]Hangzhou Institute of Medicine (HIM), Chinese Academy of Sciences, Hangzhou, Zhejiang 310018, China

[4]Ningbo Innovation Center, Zhejiang University, Ningbo 315807, China

† These authors contributed equally to this work.

*Correspondence: jingjunwu@zju.edu.cn and zhangyuhua1013@126.com

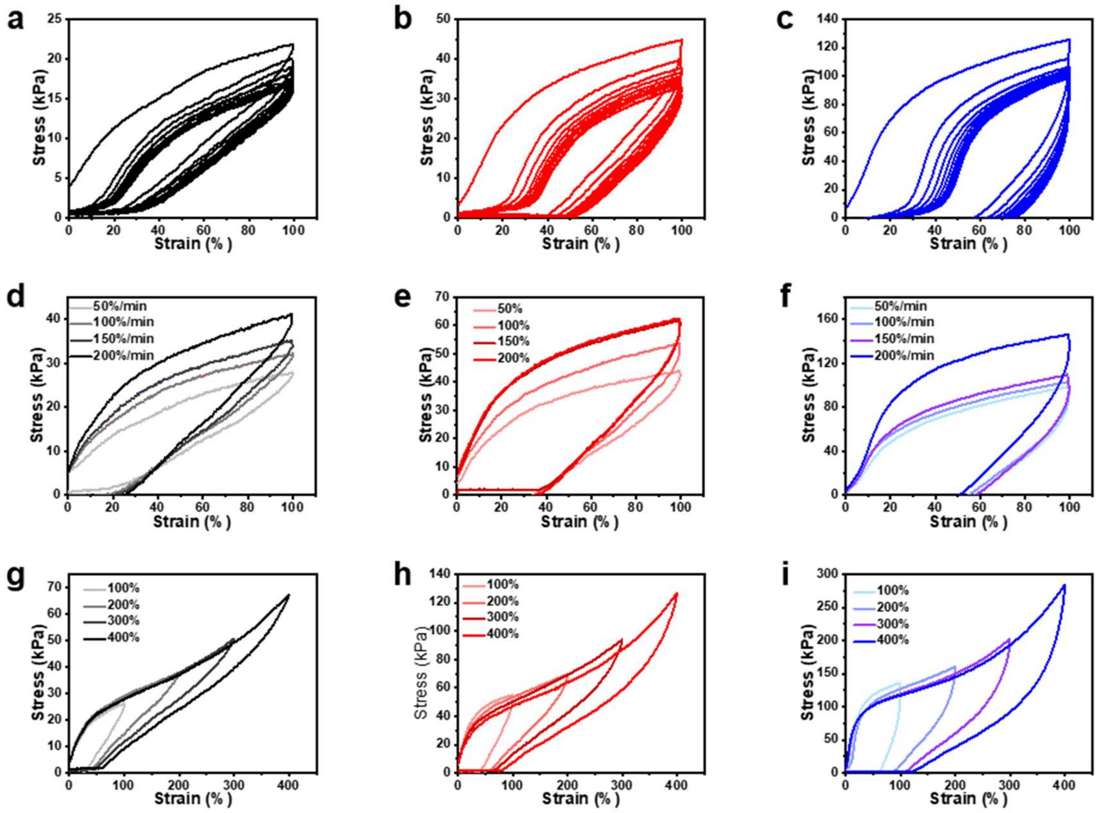

Supplementary Fig. 1 Mechanical characterization of sample ACEG-x. (Black: ACEG-0.44; Red: ACEG-0.50; Blue: ACEG-0.56) a\b\c: hysteretic curves for five times. (Strain rate: 100%/min) d\e\f: hysteretic curves for different strain rate. g/h/i: hysteretic curves for different strain. (Strain rate: 100%/min)

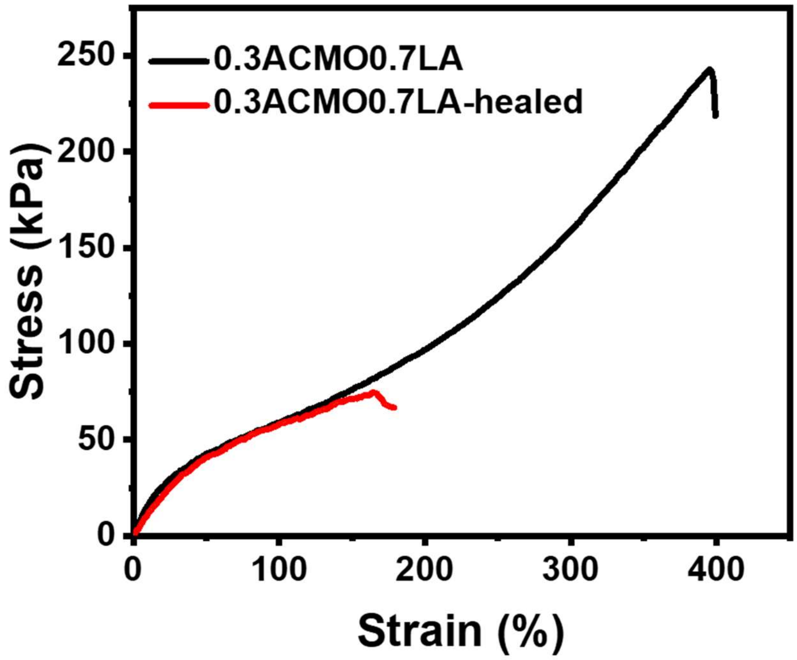

Supplementary Fig. 2 Self-healing performance of samples without hydrogen bond. Stress-strain curves of 0.3ACMO0.7LA samples before and after healing.

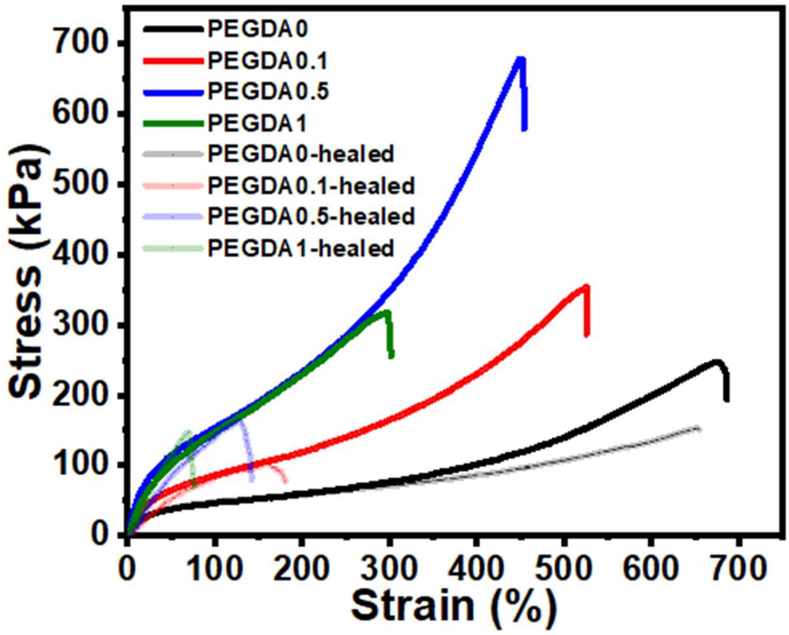

Supplementary Fig. 3 Self-healing performance of samples with different crosslinker content. Stress curves of samples before and after healing. The weight percentage of PEGDA is 0/0.1/0.5/1, respectively.

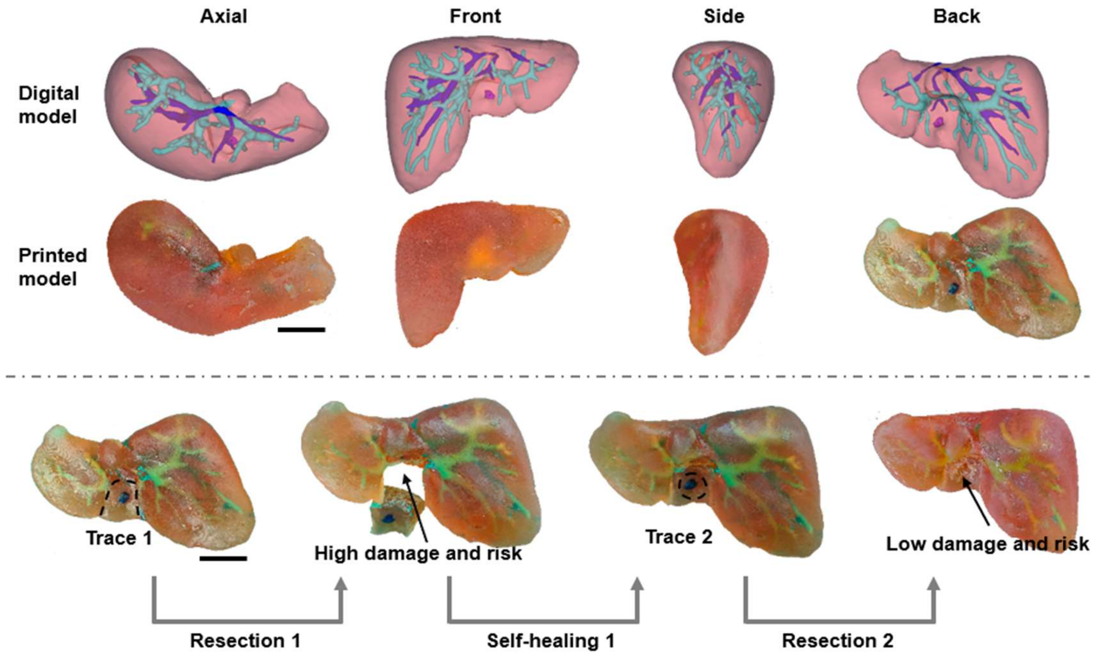

Supplementary Fig. 4 Surgical training for local hepatectomy. A small tumor in segment IV of the liver.

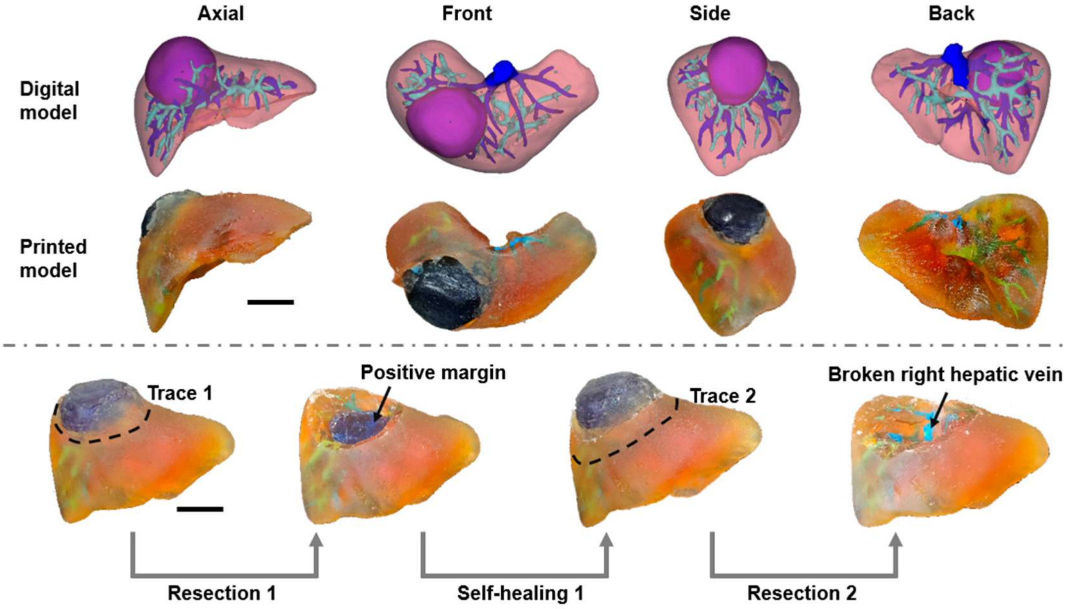

Supplementary Fig. 5 Surgical training for local hepatectomy. A big tumor in segment VIII and VII of the liver.

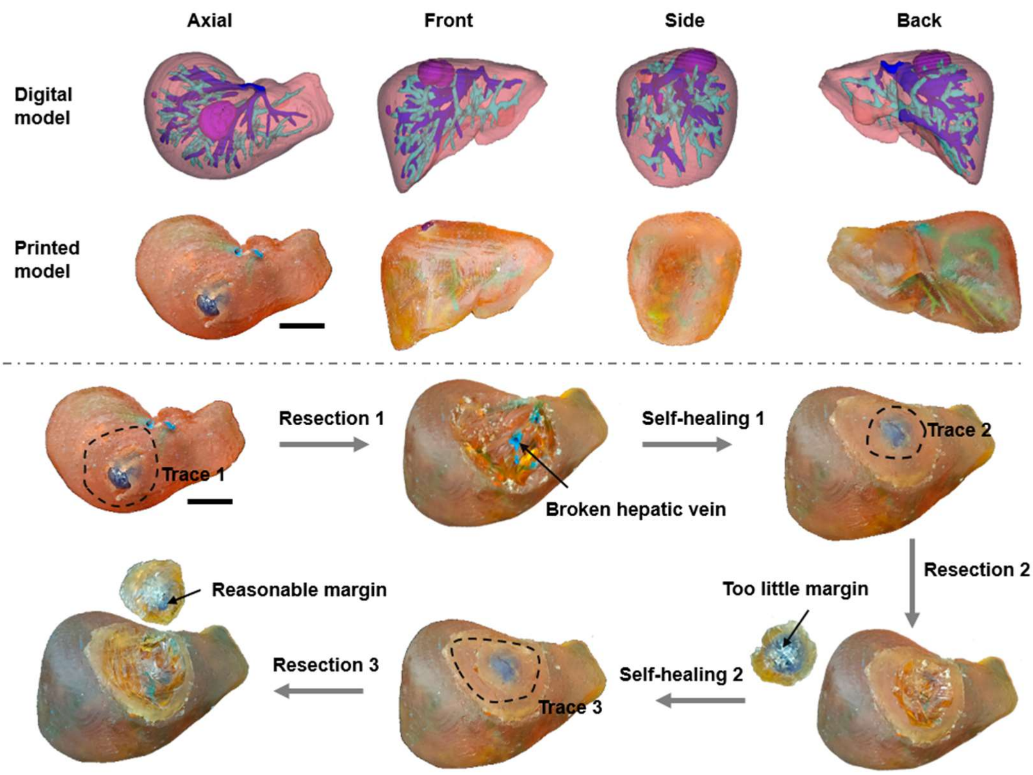

Supplementary Fig. 6 Surgical training for local hepatectomy. A tumor in segment VIII of the liver.

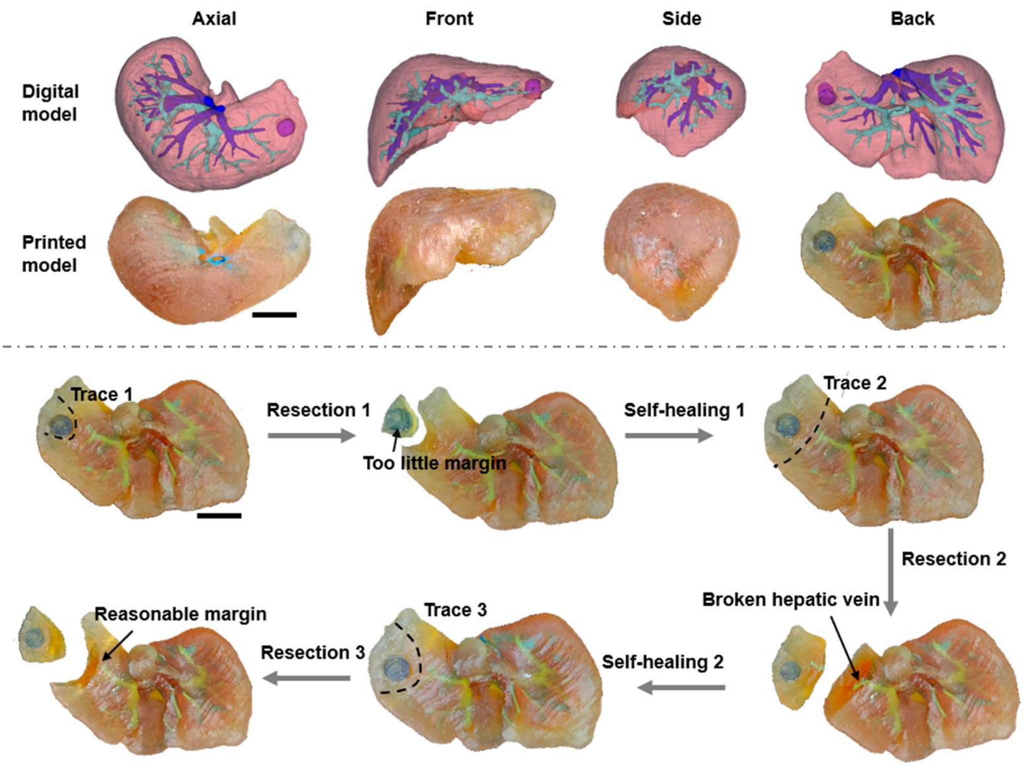

Supplementary Fig. 7 Surgical training for local hepatectomy. A small tumor in segment III of the liver.

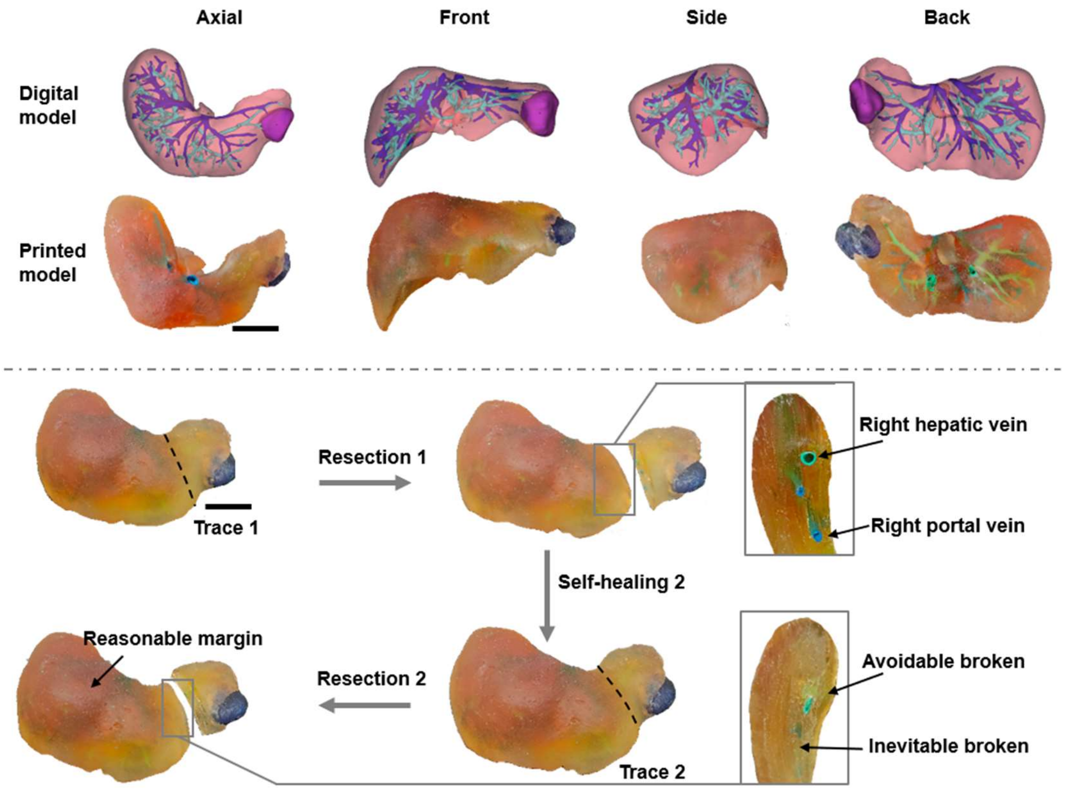

Supplementary Fig. 8 Surgical training for left hemi-hepatectomy. A tumor in segment II and III of the liver.

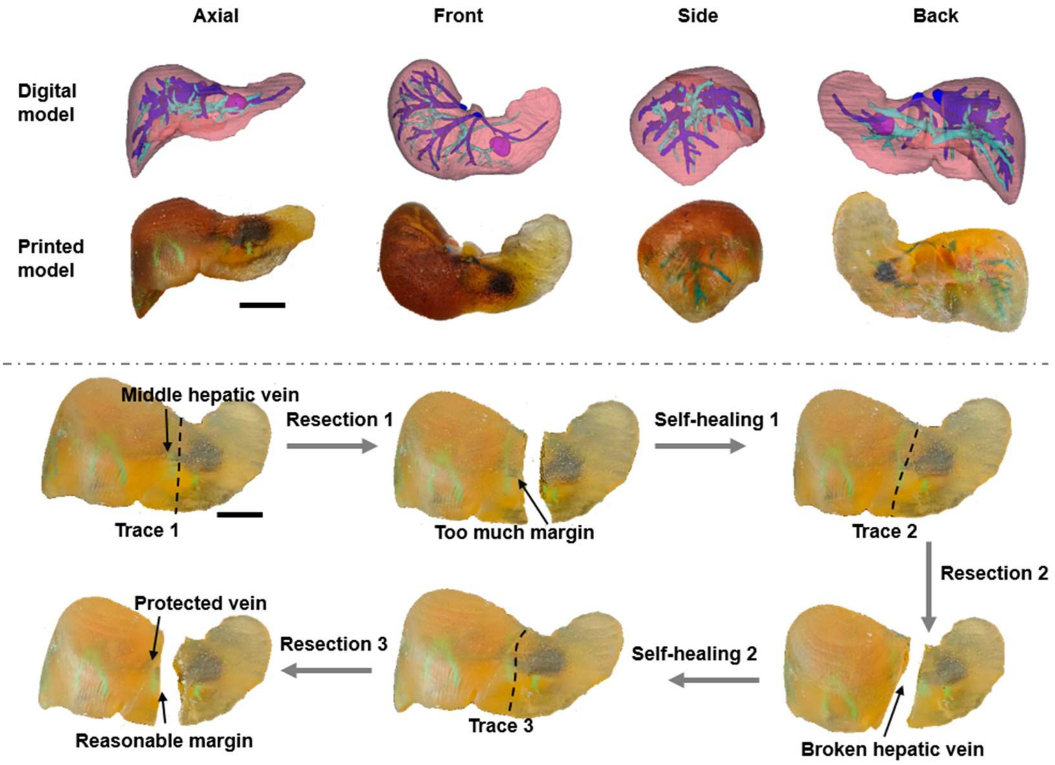

Supplementary Fig. 9 Surgical training for left hemi-hepatectomy. A tumor inside segment VI of the liver.

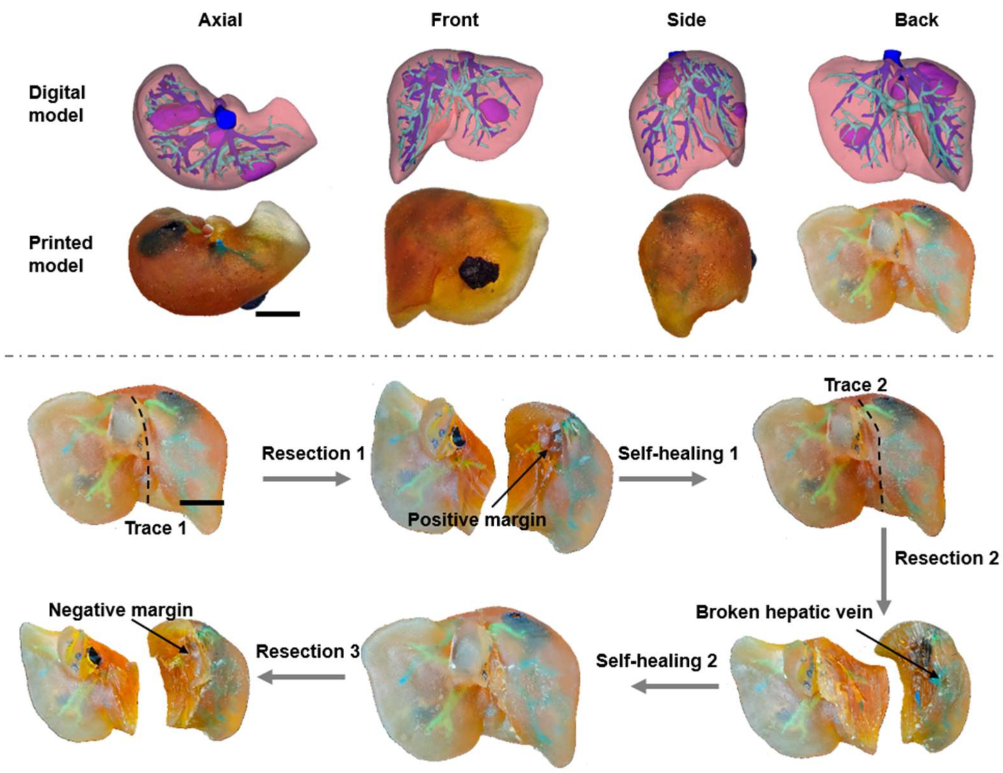

Supplementary Fig. 10 Surgical training for left hemi-hepatectomy. A tumor inside segment IV of the liver.

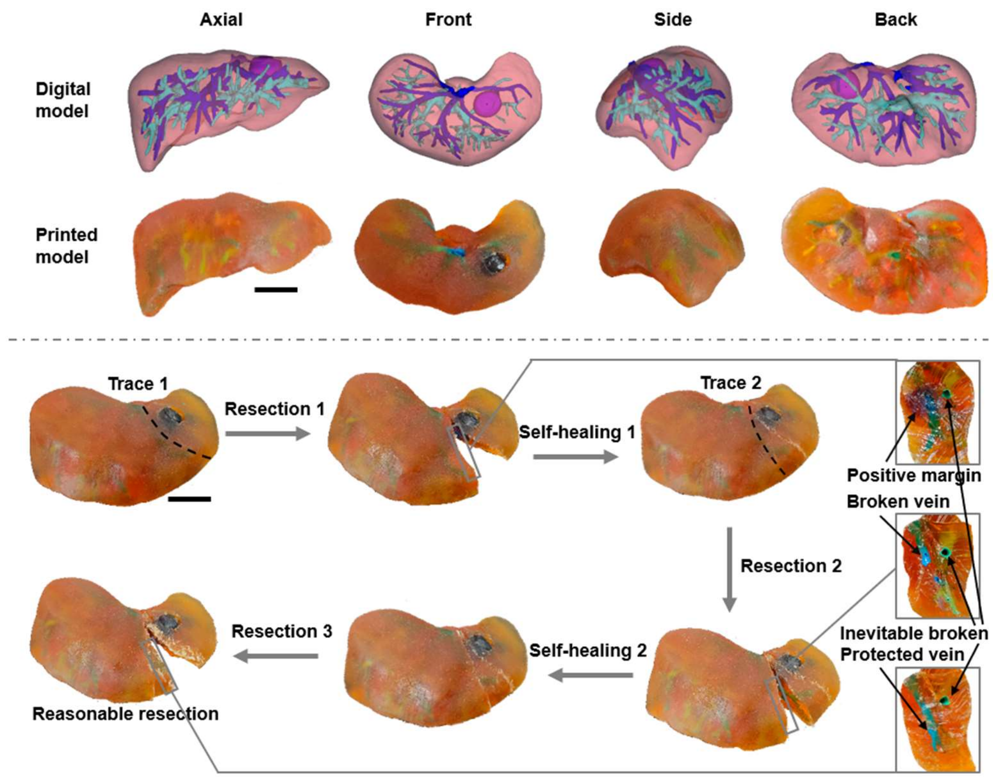

Supplementary Fig. 11 Surgical training for left hemi-hepatectomy. A tumor inside segment IV of the liver.

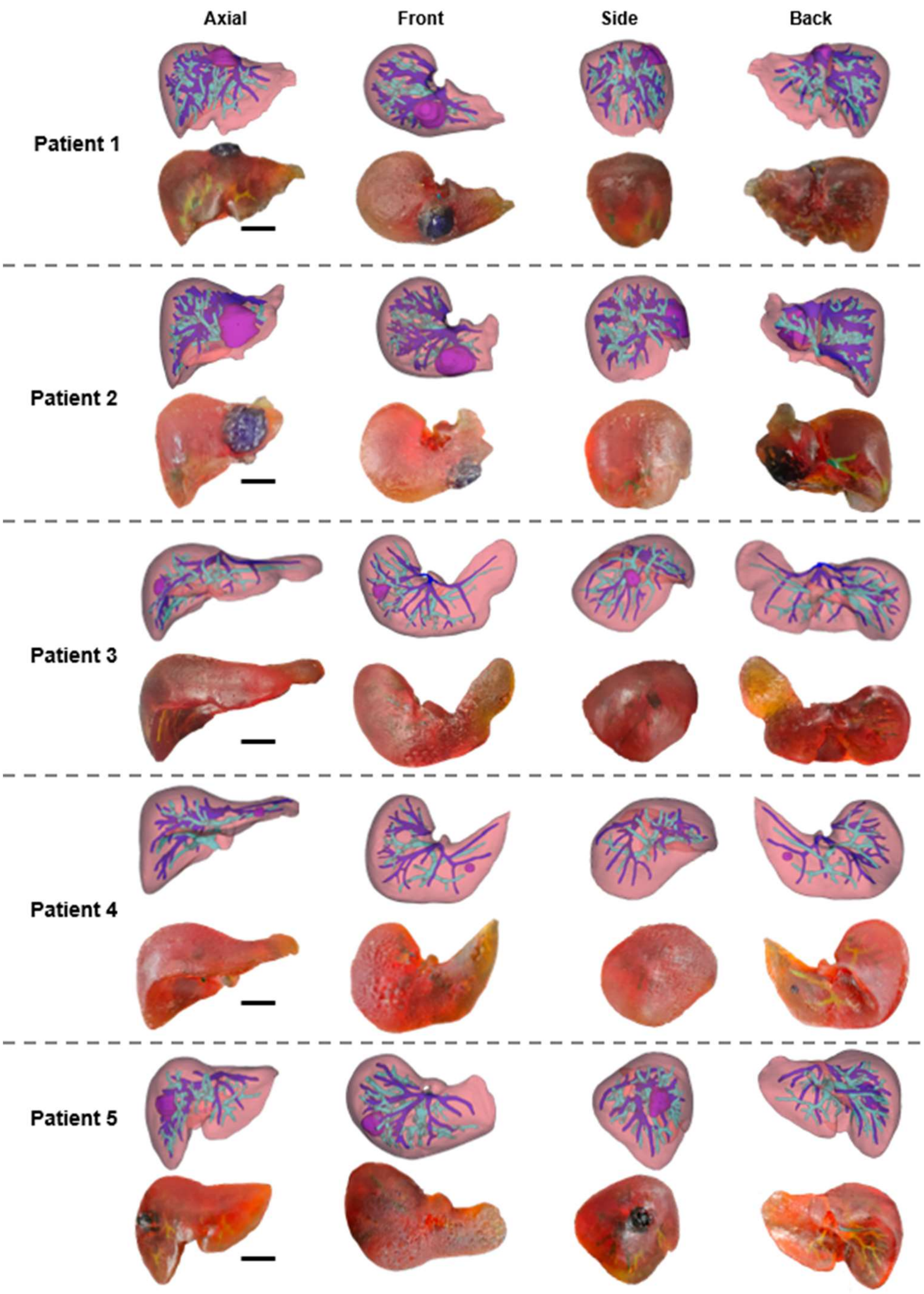

Supplementary Fig. 12 Five enrolled patients with different pathological features.

Patient ID	Sex	Age	Condition	Resection types	Blood loss (ml)	Operation time (min)	Injury of vital vascular structure	Surgical margin
1	Male	50	colorectal liver metastases	Enucleation	400	209	No	R0
2	Female	27	focal nodular hyperplasia	Left lateral hepatectomy	100	121	No	R0
3	Male	64	hepatocellular carcinoma	Segmentectomy	400	265	No	R0
4	Male	36	focal nodular hyperplasia	Left lateral hepatectomy	200	215	No	R0
5	Male	59	intrahepatic cholangiocarcinoma	Enucleation	400	388	No	R0

Supplementary Table 1 Clinical characteristics and operation outcomes of 5 patients.
