# Supplementary material for: 3D printing of self-healing personalized liver models for surgical training and preoperative planning
Source: Nat Commun. 2023 Dec 19;14:8447. doi: 10.1038/s41467-023-44324-6 (PMC10730511; doi:10.1038/s41467-023-44324-6)
Supplement: Supplementary file 3 — Reporting Summary [file 41467_2023_44324_MOESM3_ESM.pdf]

nature portfolio

Corresponding author(s): Jingjun Wu

Last updated by author(s): Dec 1, 2023

Reporting Summary

Nature Portfolio wishes to improve the reproducibility of the work that we publish. This form provides structure for consistency and transparency in reporting. For further information on Nature Portfolio policies, see our Editorial Policies and the Editorial Policy Checklist.

Statistics

For all statistical analyses, confirm that the following items are present in the figure legend, table legend, main text, or Methods section.

n/a	Confirmed	
☐	☒	The exact sample size (n) for each experimental group/condition, given as a discrete number and unit of measurement
☐	☒	A statement on whether measurements were taken from distinct samples or whether the same sample was measured repeatedly
☒	☐	The statistical test(s) used AND whether they are one- or two-sided<br>*Only common tests should be described solely by name; describe more complex techniques in the Methods section.*
☒	☐	A description of all covariates tested
☒	☐	A description of any assumptions or corrections, such as tests of normality and adjustment for multiple comparisons
☐	☒	A full description of the statistical parameters including central tendency (e.g. means) or other basic estimates (e.g. regression coefficient) AND variation (e.g. standard deviation) or associated estimates of uncertainty (e.g. confidence intervals)
☒	☐	For null hypothesis testing, the test statistic (e.g. F, t, r) with confidence intervals, effect sizes, degrees of freedom and P value noted<br>*Give P values as exact values whenever suitable.*
☒	☐	For Bayesian analysis, information on the choice of priors and Markov chain Monte Carlo settings
☒	☐	For hierarchical and complex designs, identification of the appropriate level for tests and full reporting of outcomes
☒	☐	Estimates of effect sizes (e.g. Cohen's d, Pearson's r), indicating how they were calculated

Our web collection on statistics for biologists contains articles on many of the points above.

Software and code

Policy information about availability of computer code

Data collection	No software was used.
Data analysis	Data analysis and plot was processed by Origin 2023b. Digital liver models were built by Yorktal Digital Medical Imaging. STL liver models were processed by Materialise Magics 21.0.

For manuscripts utilizing custom algorithms or software that are central to the research but not yet described in published literature, software must be made available to editors and reviewers. We strongly encourage code deposition in a community repository (e.g. GitHub). See the Nature Portfolio guidelines for submitting code & software for further information.

Data

Policy information about availability of data

All manuscripts must include a data availability statement. This statement should provide the following information, where applicable:

- Accession codes, unique identifiers, or web links for publicly available datasets
- A description of any restrictions on data availability
- For clinical datasets or third party data, please ensure that the statement adheres to our policy

The authors declare that the data supporting the findings of this study are available within the paper and its Supplementary Information files. Additional data are available from the corresponding author upon request. Source data are provided as a Source Data file. Source data are provided with this paper.

Research involving human participants, their data, or biological material

Policy information about studies with human participants or human data. See also policy information about sex, gender (identity/presentation), and sexual orientation and race, ethnicity and racism.

Reporting on sex and gender	Sex information (male or female) for the 5 human participants was listed in Supplementary Table 1. The findings of our study are applicable to both sexes. Sex was not a factor considered in the study design. Gender identification was determined based on self-reporting. Consent has been obtained for the sharing of individual-level data, but this data was deidentified when presented in the supplementary information.
Reporting on race, ethnicity, or other socially relevant groupings	Race, ethnicity, or other socially relevant groupings are unrelated to the study.
Population characteristics	The covariate-relevant population characteristics of the human research participants were listed in supplementary Table 1.
Recruitment	The participants were recruited from patients admitted to the Hepatopancreatobiliary Surgery Department of Zhejiang Cancer Hospital requiring liver resection treatment. All eligible patients according to our inclusion and exclusion criteria were provided with information about our trial, and they made an independent decision on whether or not to participate in the study. No self-selection bias or other biases present.
Ethics oversight	The Institutional Ethical Review Board of Zhejiang Cancer Hospital.

Note that full information on the approval of the study protocol must also be provided in the manuscript.

Field-specific reporting

Please select the one below that is the best fit for your research. If you are not sure, read the appropriate sections before making your selection.

☒ Life sciences ☐ Behavioural & social sciences ☐ Ecological, evolutionary & environmental sciences

For a reference copy of the document with all sections, see nature.com/documents/nr-reporting-summary-flat.pdf

Life sciences study design

All studies must disclose on these points even when the disclosure is negative.

Sample size	No sample-size calculation was performed. It is important to note that this investigation is preliminary and exploratory in nature, with the primary objective of assessing the viability of 3D printed liver models for surgical training and preoperative planning. This project differs from traditional randomized controlled trials (RCTs) or cohort studies. In this study, our participant pool consisted of only five individuals, and based on the current data, we are confident that the feasibility of utilizing 3D printed liver models for the intended purposes has been substantiated.
Data exclusions	No data were excluded from the analyses.
Replication	Our primary objective is to conduct a preliminary investigation into the potential of 3D printed models as tools for surgical training and preoperative planning. The entire workflow, encompassing 3D reconstruction, 3D printing, try-and-error simulation surgery, and actual surgery, has been successfully replicated in five distinct patients. All replication attempts were successful, and five patients were discharged without complications.
Randomization	Our study comprised only one group of patients, and randomization was not relevant to your study.
Blinding	Our study comprised only one group of patients, and blinding was not relevant to your study.

Reporting for specific materials, systems and methods

We require information from authors about some types of materials, experimental systems and methods used in many studies. Here, indicate whether each material, system or method listed is relevant to your study. If you are not sure if a list item applies to your research, read the appropriate section before selecting a response.

Materials & experimental systems

n/a	Involved in the study
[x]	[ ] Antibodies
[x]	[ ] Eukaryotic cell lines
[x]	[ ] Palaeontology and archaeology
[x]	[ ] Animals and other organisms
[ ]	[x] Clinical data
[x]	[ ] Dual use research of concern
[x]	[ ] Plants

Methods

n/a	Involved in the study
[x]	[ ] ChIP-seq
[x]	[ ] Flow cytometry
[x]	[ ] MRI-based neuroimaging

Clinical data

Policy information about clinical studies

All manuscripts should comply with the ICMJEguidelines for publication of clinical research and a completedCONSORT checklist must be included with all submissions.

Clinical trial registration	NCT06006338.
Study protocol	The full trial protocol can be accessed as we have uploaded the English version as a separate file in the manuscript system.
Data collection	Data were obtained from the Electronic Medical System of Zhejiang Cancer Hospital, and patient recruitment took place from September 2023 to October 2023. The first case was collected on September 15 2023 and the last was October 9 2023.
Outcomes	The pre-defined primary and secondary outcomes were measured using data extracted from the Electronic Medical System of Zhejiang Cancer Hospital. These outcomes were assessed by experienced surgeons.<br>Primary Outcome Measure:<br>(1) R0 resection rate: The proportion of patients who achieved pathological negative surgical margin.<br>Secondary Outcome Measure:<br>(2) Injury of vital vascular structures: The Injury of vital vascular structures, such as portal veins or hepatic veins.<br>(3) Operation time: Time length (minute) between the beginning and the end of the surgery.<br>(4) Blood loss: The amount of lost blood (ml) in the operation.

Plants

Seed stocks	*Report on the source of all seed stocks or other plant material used. If applicable, state the seed stock centre and catalogue number. If plant specimens were collected from the field, describe the collection location, date and sampling procedures.*
Novel plant genotypes	*Describe the methods by which all novel plant genotypes were produced. This includes those generated by transgenic approaches, gene editing, chemical/radiation-based mutagenesis and hybridization. For transgenic lines, describe the transformation method, the number of independent lines analyzed and the generation upon which experiments were performed. For gene-edited lines, describe the editor used, the endogenous sequence targeted for editing, the targeting guide RNA sequence (if applicable) and how the editor was applied.*
Authentication	*Describe any authentication procedures for each seed stock used or novel genotype generated. Describe any experiments used to assess the effect of a mutation and, where applicable, how potential secondary effects (e.g. second site T-DNA insertions, mosiacism, off-target gene editing) were examined.*
